# Pre-epithelialized cryopreserved tracheal allograft for neo-trachea flap engineering

**DOI:** 10.3389/fbioe.2023.1196521

**Published:** 2023-05-05

**Authors:** Ning Zeng, Youbai Chen, Yewen Wu, Mengqing Zang, Rene D. Largo, Edward I. Chang, Mark V. Schaverien, Peirong Yu, Qixu Zhang

**Affiliations:** Department of Plastic Surgery, The University of Texas MD Anderson Cancer Center, Houston, TX, United States

**Keywords:** tracheal tissue engineering, tracheal cryopreservation, partial decellularization, pre-epithelialization, vascularization

## Abstract

**Background:** Tracheal reconstruction presents a challenge because of the difficulty in maintaining the rigidity of the trachea to ensure an open lumen and in achieving an intact luminal lining that secretes mucus to protect against infection.

**Methods:** On the basis of the finding that tracheal cartilage has immune privilege, researchers recently started subjecting tracheal allografts to “partial decellularization” (in which only the epithelium and its antigenicity are removed), rather than complete decellularization, to maintain the tracheal cartilage as an ideal scaffold for tracheal tissue engineering and reconstruction. In the present study, we combined a bioengineering approach and a cryopreservation technique to fabricate a neo-trachea using pre-epithelialized cryopreserved tracheal allograft (ReCTA).

**Results:** Our findings in rat heterotopic and orthotopic implantation models confirmed that tracheal cartilage has sufficient mechanical properties to bear neck movement and compression; indicated that pre-epithelialization with respiratory epithelial cells can prevent fibrosis obliteration and maintain lumen/airway patency; and showed that a pedicled adipose tissue flap can be easily integrated with a tracheal construct to achieve neovascularization.

**Conclusion:** ReCTA can be pre-epithelialized and pre-vascularized using a 2-stage bioengineering approach and thus provides a promising strategy for tracheal tissue engineering.

## Graphical Abstract

**Figure:**
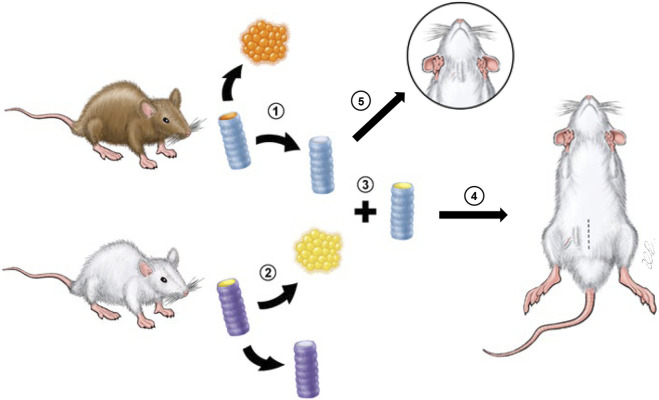
Scheme of the experimental design. 1, CTA derived from donor Brown Norway rats was de-epithelialized to create DeCTA. 2, RECs were isolated from syngeneic recipient Lewis rats. 3, DeCTA was pre-epithelialized to create ReCTA. 4, In the heterotopic implantation model, ReCTA was subcutaneously implanted into the groin area, and an adipose tissue flap pedicled by superficial epigastric blood vessels was used for neovascularization. 5, In the orthotopic implantation model, DeCTA was implanted into the neck to reconstruct a 4-ring tracheal defect.

## Introduction

Tracheal defects can result from congenital disease, trauma, tumors, or the treatment thereof (Grillo and Mathisen, 1990; Gandhi et al., 1999; Rendina et al., 1999; Mitchell et al., 2001; American Cancer Society, 2002; Grillo, 2004). Tracheal defects as long as 5 cm can be closed primarily, but anastomotic complications and mortality rates increase as the defect length increases (Mitchell et al., 2001). Longer defects that cannot be closed primarily require reconstruction with a tracheal conduit. In tracheal reconstruction, the rigidity of the trachea must be maintained to ensure an open lumen, and the luminal lining must be intact to secrete mucus and protect against infection. These unique requirements have challenged reconstructive surgeons for decades. Efforts to reconstruct longer and/or circumferential tracheal defects have been largely unsuccessful. Therefore, patients with extensive tracheal involvement typically are not surgical candidates, or they may require total laryngectomy and possibly mediastinal tracheostomy, which is associated with a high mortality rate. Obviously, successful tracheal reconstruction would extend the surgical indications for cure and offer patients a good quality of life.

Tracheal reconstruction has evolved from the simple implantation of prosthetic materials to the use of autologous soft tissue flaps with supporting materials to the use of entirely tissue-engineered tracheas (Pan et al., 2022a; Pan et al., 2022b; Tsou et al., 2023; Hiwatashi et al., 2022; Liu CS et al., 2022). Although prosthesis materials have evolved from stainless steel to porous materials with better biocompatibility, a prosthesis alone always fails to re-epithelialize, leading to infection and rejection (Cull et al., 1990; Teramachi et al., 1997; Matloub and Yu, 2006). Tracheal repair with a segment of small bowel with or without a cartilage graft has been attempted in dogs and goats, but these reconstructions failed or resulted in high mortality rates (up to 70%) because of airway collapse and obstruction due to mucus production (Letang et al., 1990; Mukaida et al., 1998). Other autologous tissues, such as free periosteal, muscular, esophageal, bronchial, and aortic grafts, have also been used for tracheal reconstruction, but merely to maintain airway patency (Letang et al., 1990; Cavadas, 1998; Matloub and Yu, 2006). Chemically fixed cadaveric tracheal allografts have been used to repair short, partial (i.e., noncircumferential) tracheal defects caused by benign stenosis (Cull et al., 1990; Anderl and Haid, 2005), but such reconstructions are prone to resorption and loss of rigidity, leading to airway collapse, and obviously are not suitable for extensive defects (Beldholm et al., 2003).

To date, only a few clinical applications of large-scale tracheal reconstruction have been relatively successful (Genden and Laitman, 2023). On the basis of a series of animal experiments (Matloub and Yu, 2006; Zang et al., 2010), we developed an approach in which an autologous skin flap is used to line the lumen of a prosthesis which is used as a support scaffold for the repair of large tracheal defects, and we investigated its use in 7 cancer patients. However, this approach is very complicated; it is suitable only for strictly selected patients, and it should be attempted only by skillful microsurgeons (Yu et al., 2006; 2011; Ghali et al., 2015). Macchiarini et al. (2008) performed the first reconstruction with a tissue-engineered trachea [a completely decellularized tracheal scaffold seeded with the patient’s own bone marrow (BM)-derived stem cells and respiratory epithelial cells (RECs)] in 2008 (Macchiarini et al., 2008). However, the uncertain cartilage regeneration in this approach and the issue around the clinical sets makes it very difficult to implement (Vogel, 2013; Delaere and Van Raemdonck, 2014; Gonfiotti et al., 2014; Kojima and Vacanti, 2014). In other studies, we found that stem cells seeded on the surface of an acellular tracheal matrix scaffold appeared to have hardly migrated into the cartilage lacunae or differentiated there (Zang et al., 2012; Zang et al., 2013), thus, the scaffold may require some modification to improve the migration of chondrocytes across the collagen matrix boundary into the center of the scaffold (Xu et al., 2017). Realizing the pitfalls of using a completely decellularized tracheal scaffold for tracheal tissue engineering, Jungebluth et al. (2011) switched to using a synthetic tube-type prosthetic scaffold for clinical tracheal reconstruction. Unfortunately, most of the patients who received the synthetic tracheas died shortly after their implantation.

The optimal method of tracheal reconstruction would be to replace the native trachea with another trachea via composite tissue allotransplantation. However, traditional organ transplantation requires life-long immunosuppression, whose potential severe side effects, including opportunistic infections, end-organ toxicity, and neoplasms, render the technique unsuitable for many applications. A breakthrough in clinical tracheal allotransplantation without immunosuppression was achieved in 2010, when Delaere et al. (2010) used a 2-stage microsurgical approach to orthotopically transfer a vascularized tracheal flap comprising a fresh viable cartilage allograft covered with a buccal mucosa isograft. The success of the case indicated that the epithelium, perichondrium, and mucosal and submucosal glands are the major antigenic structures of the trachea (and thus responsible for transplant rejection), whereas tracheal cartilage is an immune-privileged tissue; its avascularity and dense matrix enable it to evade host immune recognition (Sykes, 2010; Huey et al., 2012). However, recipient cells are slow to repopulate the mucosal lining, and this has hindered the widespread clinical use of the approach (Delaere et al., 2012). Nevertheless, the experience of Delaere et al. (2012) demonstrates that in orthotopically implanted fresh tracheal allograft (FTA), the cartilage can have restored function after its re-epithelialization by recipient buccal mucosa and requires no immunosuppression.

Inspired by the clinical trials of Delaere et al. (2010) (Sykes, 2010; Delaere et al., 2012; Huey et al., 2012), researchers have started subjecting tracheal allografts to “partial decellularization” (in which only the epithelium and its antigenicity are removed), rather than complete decellularization, to maintain the tracheal cartilage as an ideal scaffold for tracheal tissue engineering and reconstruction. The transplantation of cryopreserved tracheal allografts (CTAs) has been investigated for decades (Sotres-Vega et al., 2006; Nakanishi, 2009), but most of these allografts (Nakanishi, 2009), as well as many partially decellularized scaffolds (Hysi et al., 2014; Hysi et al., 2015; Den Hondt et al., 2016; De Wolf et al., 2017; Den Hondt et al., 2017; Cui et al., 2021; Wang et al., 2021), were not pre-epithelialized with real RECs. Therefore, only the short allografts achieved satisfactory results *in vivo*, whereas the longer ones failed to maintain patency, mostly because of proliferating fibroblasts that originated from the lamina propria and was caused by a lack of proper REC coverage (Hysi et al., 2015).

Combining a bioengineering approach and a cryopreservation technique, we aimed to fabricate a neo-trachea using pre-epithelialized CTA (ReCTA). We hypothesized that, after its vascularization *in vivo*, our engineered neo-trachea flap can evade host immune recognition without the need for immunosuppression and retain both cartilage and epithelium function. To test our hypothesis, we first partially decellularized CTA, removing only the epithelium while preserving chondrocytes. We then cultured RECs in the lumen of the tracheal scaffold for pre-epithelialization. We then heterotopically and orthotopically implanted the engineered neo-trachea into rodent models to examine its viability and rigidity (Graphical Abstract). The findings of the present study may inform the development of a platform for designing and fabricating neo-trachea flap that can be used to reconstruct longer tracheal defects.

## Materials and methods

### Tracheal cryopreservation and partial decellularization

All procedures were approved by MD Anderson’s Institutional Animal Care and Use Committee and met all requirements of the Animal Welfare Act.

We used 8- to 10-week-old male Brown Norway rats (Harlan Laboratories, Indianapolis, IN; *n* = 21) weighing 250 g each to provide donor tracheas for tracheal allograft scaffolds. Each mouse’s trachea was harvested and processed under sterile conditions. The entire length of the trachea was harvested and carefully stripped of overlying soft tissue. The trachea was then divided into 2 segments of approximately 8 rings each and frozen as described previously (Sotres-Vega et al., 2006). Briefly, the samples were immersed in protective medium (DMEM containing 20% FBS, 10% DMSO, 0.1 M trehalose, 0.1 M sucrose, 100 μg/mL penicillin, and 100 μg/mL streptomycin) and frozen at 4°C for 60 min, then frozen to −80°C at a controlled rate of −1°C/min, and finally stored at −196°C for 1 month. The samples were thawed in a 37°C water bath and rinsed in DMEM for further use. For decellularization, the samples were washed in sterile phosphate-buffered saline (PBS) at 4°C (except as indicated otherwise) with agitation at 80 rpm for 2–12 h. The resultant de-epithelialized CTA (DeCTA) samples were then characterized using the following methods.

### Immunohistochemical analysis

FTA, CTA, and DeCTA were fixed in 10% formalin, embedded in paraffin, and sliced into 5-µm sections. The sections were deparaffinized, rehydrated, washed in distilled water, and mounted on slides. The sections were subjected to histological hematoxylin and eosin (H&E) staining, 4′-6-diamidino-2-phenylindole (DAPI) staining, Masson trichrome staining, and Safranin O staining. For immunohistochemical staining, the sections were placed in antigen retrieval citrate buffer (Biogenex, Fremont, CA) in a steamer at 95°C for 10 min. Endogenous peroxidases were blocked by incubation with Peroxide Block (Innogenex, San Ramon, CA), and nonspecific binding was blocked with normal goat serum (Vector Laboratories, Burlingame, CA). The sections were incubated overnight with primary antibodies against MHC-1, MHC-II, OX62, collagen II, and collagen IV (all at 1:200; Abcam, Cambridge, MA) at 4°C. The sections were washed, and biotinylated secondary antibody was applied for 30 min, followed by treatment with streptavidin-horseradish peroxidase complex (Vectastain ABC Kit, Vector Laboratories) and diaminobenzidine solution (DAB Kit, Vector) and counterstaining with hematoxylin. The sections were dehydrated, mounted, and imaged using an IX71 microscope (Olympus, Center Valley, PA).

### Sulfated glycosaminoglycan content

The sulfated glycosaminoglycan (GAG) content of native tissues and decellularized samples was quantified using an Alcian blue colorimetric assay kit (sGAG Dye Binding Assay, ALPCO, Salem, NH). Samples were lyophilized, papain-digested, and then incubated with Alcian blue dye. Absorbance of the samples at 600–620 nm was measured with a DU 730 UV/Vis Spectrophotometer (Beckman Coulter) using chondroitin sulfate (Sigma, St. Louis, MO) as the standard. GAG content was normalized to the initial dry weight of the samples.

### Scanning electron microscopy

For scanning electron microscopy (SEM), samples were fixed in a solution containing 3% glutaraldehyde plus 2% paraformaldehyde in 0.1 M cacodylate buffer, pH 7.3, washed with 0.1 M cacodylate buffer, pH 7.3, post-fixed with 1% cacodylate-buffered osmium tetroxide, washed with 0.1 M cacodylate buffer, and then washed with distilled water. Next, the samples were sequentially treated with Millipore-filtered 1% aqueous tannic acid, washed with distilled water, treated with Millipore-filtered 1% aqueous uranyl acetate, and then rinsed thoroughly with distilled water. The samples were dehydrated with a graded series of increasing concentrations of ethanol, transferred to a graded series of increasing concentrations of hexamethyldisilazane, and air-dried overnight. The samples were mounted on double-stick carbon tabs (Ted Pella, Inc., Redding, CA), which were previously mounted on aluminum specimen mounts (Electron Microscopy Sciences, Fort Washington, PA). The samples received a 25-nm coating of platinum alloy under vacuum using a Balzer MED 010 evaporator (Technotrade International, Manchester, NH) and were then immediately flash carbon-coated under vacuum. The samples were transferred to a desiccator for examination at a later date. Samples were imaged and examined using a JSM-5910 scanning electron microscope (JEOL USA, Inc., Peabody, MA) at an accelerating voltage of 5 kV.

### Transmission electron microscopy

For transmission electron microscopy (TEM), samples were fixed with a solution containing 3% glutaraldehyde plus 2% paraformaldehyde in 0.1 M cacodylate buffer, pH 7.3, washed in 0.1 M sodium cacodylate buffer, treated with 0.1% Millipore-filtered cacodylate-buffered tannic acid, post-fixed with 1% buffered osmium, and then stained *en bloc* with 1% Millipore-filtered uranyl acetate. The samples were dehydrated in increasing concentrations of ethanol, filtered, and embedded in LX-112 medium. The samples were polymerized in a 60°C oven for approximately 3 days. Ultrathin sections were cut in an Ultracut microtome (Leica, Deerfield, IL), stained with uranyl acetate and lead citrate in an EM Stainer (Leica), and examined using a JEM 1010 transmission electron microscope (JEOL USA, Inc.) at an accelerating voltage of 80 kV. Digital images were obtained using an AMT Imaging System (Advanced Microscopy Techniques Corp., Danvers, MA).

### Contact assay

To determine whether DeCTA is cytotoxic, we performed a contact assay in which the scaffold was co-cultured with BM-derived mesenchymal stem cells (BM-MSCs). Briefly, we isolated BM-MSCs from 8- to 10-week-old male Lewis rats (Harlan Laboratories, Indianapolis, IN) weighing 250 g each using our established protocol (Zang et al., 2012; Zang et al., 2013). BM-MSCs were cultured in medium and harvested at passage 3. The cells were seeded in a petri dish, and then DeCTA was placed in the same petri dish. On day 3, the cells were washed with PBS 3 times. Then, the cells were exposed to 2 μM calcein AM and ethidium homodimer-1 using the LIVE/DEAD Viability/Cytotoxicity Kit (Molecular Probes, Carlsbad, CA) according to the manufacturer’s instructions. Briefly, the samples were washed with PBS 3 times and then incubated with the staining reagents in PBS for 30 min at 37°C before the staining solution was replaced. The samples were washed with PBS 3 times before they were viewed using an IX81 confocal microscope (Olympus).

### Chondrocyte isolation and culture

Chondrocytes were isolated from DeCTA using methods we described previously (Zang et al., 2011; Zang et al., 2013). Briefly, cartilage sheets from 3 DeCTA samples were minced into pieces of approximately 1 mm^3^ and digested in 0.1% collagenase type II (Worthington Biochemical Corp., Lakewood, NJ) in DMEM with 4.0 g/L glucose (Hyclone, Waltham, MA) on a shaker for 21 h at 37°C. The resultant cell suspension was centrifuged at 200 g for 8 min, and the cell pellet was resuspended in growth medium consisting of DMEM with 4.0 g/L glucose supplemented with 10% FBS (Atlanta Biologicals, Lawrenceville, GA) and 1% antibiotics (100 U/mL penicillin G, 100 μg/mL streptomycin sulfate, and 250 ng/mL amphotericin B; Hyclone). Then, the cells were plated at a density of 10,000 cells/cm^2^ and incubated at 37°C with media changes every 3 days. At 80% confluence, the cells were detached with 0.05% trypsin-EDTA (Gibco, Carlsbad, CA), plated in 2-well chamber slides for culturing, and stained with Alcian blue (Sigma).

Chondrocyte proliferation was measured with CellQuanti-MTT Cell Viability Assay Kits (BioAssay Systems, Hayward, CA) according to the manufacturer’s instructions. Chondrocytes isolated from DeCTA were placed in 96-well tissue culture plates at a density of 10,000 cells/cm^2^ in 100 μL of medium. The cells were washed and treated with a 1:5 ratio of 3(4,5-dimethylthiazol-2Yl)-2,5-diphenyltetrazolium bromide (MTT) to culture media 1, 3, and 7 days after seeding. Following incubation at 37°C for 2 h, MTT was taken up by active cells and reduced inside the mitochondria to insoluble purple formazan granules. The cells were subsequently lysed, and the formazan granules were solubilized in dimethyl sulfoxide on a shaker. The supernatant was transferred to fresh 96-well plates to reduce background contamination, and the optical density was read at 540 nm using a photometric plate reader (BioTek, Oklahoma City, OK).

### REC isolation and culture

Tracheal RECs were isolated from Lewis rats using methods we described previously (Zang et al., 2012; Zang et al., 2013). Briefly, RECs were dissociated from the tracheal mucosal lining with 2 mg/mL pronase (Roche Applied Science, Indianapolis, IN) in Ham’s F-12 medium overnight (12 h) at 4°C. Cells were collected by centrifugation at 500 g for 10 min. Cell pellets were re-suspended in DMEM/Ham’s F-12 medium (Sigma) supplemented with 10 μg/mL insulin (Sigma), 0.1 μg/mL hydrocortisone (Sigma), 0.1 μg/mL cholera toxin (Sigma), 5 μg/mL transferrin (BD, Franklin Lakes, NJ), 50 μM phosphoethanolamine (Sigma), 80 μM ethanolamine (Sigma), 25 ng/mL epidermal growth factor (BD), 70 μg/mL bovine pituitary extract (United States Biological, Swampscott, MA), 3 mg/mL bovine serum albumin (Sigma), and 5 nM retinoic acid (Sigma). Epithelial cells at passage 0 were centrifuged with a Shandon Cytospin (GMI, Inc., Ramsey, MN) and stained with antibodies against cytokeratin peptide 17, beta tubulin IV, mucin 5AC, and P63 (all at 1:200; Sigma) to identify their components. We found that the percentage of cells expressing the markers of rat respiratory tract epithelial differentiation decreased during subculture. Because most cells became fibroblast-like cells in passage 2, we used only freshly isolated RECs for cell seeding in the ensuing experiments.

### Creation of ReCTA

RECs were seeded into the lumen of DeCTA at a density of 1 × 10^5^ cells/cm^2^. Acland artery clamps were used to seal the lumen ends, and DeCTA was cultivated in epithelial cell growth medium. Twenty-four hours later, the pre-epithelialized DeCTA (i.e., ReCTA) was stained with calcein AM and ethidium homodimer-1 using the LIVE/DEAD Viability/Cytotoxicity Kit as described above. SEM was used to assess cell morphology.

### *In vivo* evaluation of ReCTA in a heterotopic implantation model

To assess the neovascularization and survival of ReCTA *in vivo*, we heterotopically implanted ReCTA and other tracheal grafts into 8- to 10-week-old male Lewis rats. Rats were anesthetized and maintained with isoflurane (0.5%–2%, 3–5 L/min) and oxygen during surgery.

In the heterotopic implantation model, rats received FTA, CTA, DeCTA, ReCTA, or fresh isograft trachea (FIT) (*n* = 6/group). FTA, CTA, DeCTA, and ReCTA were allografts from Brown Norway rats. FIT was from Lewis rats. Both FTA and FIT were rinsed with PBS to remove additional mucus and blood and then preserved in PBS. CTA was quickly thawed in a 37°C water bath, simply rinsed with PBS to remove cryoprotectants, and then preserved in PBS. DeCTA was prepared as described above. ReCTA was prepared as described above; briefly, RECs isolated from one Lewis rat trachea were suspended with REC growth media and injected into the lumen of one DeCTA scaffold (8 rings), and then both ends of the scaffold were sealed with Acland arterial clamps, and the scaffold was implanted immediately to avoid longer ischemia. The warm ischemia time was within 4 h for all grafts before implantation. In each animal, one graft was carefully implanted under the fat pad of the adipose tissue flap pedicled by the superficial epigastric blood vessel in the groin area. Animals were monitored for clinical signs of inflammation or rejection for 30 days and then humanely killed for graft explantation.

### *In vivo* evaluation of DeCTA in an orthotopic implantation model

In the orthotopic implantation model, a 5 mm section of trachea (3-4-ring) was removed from each Lewis rat and replaced with a tracheal graft of the same size. Rats received FIT or DeCTA (*n* = 5/group). Rats were anesthetized with IM ketamine/xylazine. A 2 cm midline cervical incision was made in line with the trachea. The strap muscles were divided and the entire trachea was exposed. The vascular structures and the recurrent laryngeal nerves were sharply dissected away from the trachea. A 3-4-ring segment was removed and the graft was interposed between the recipient tracheal defects and anastomosed after ensuring adequate hemostasis and clean tracheal edges (Supplementary Figure S1). Animals were monitored for clinical signs of respiratory distress for 3 months and then humanely killed for graft explantation.

### Immunohistochemical analysis of *in vivo* explants

Specimens cut from the centers of the heterotopic explants were fixed in 10% neutral buffered formalin and embedded in paraffin. Sections cut from the paraffin-embedded samples were subjected to histological H&E staining and immunohistochemical staining with antibodies against CD68, CD4, and CD8 (all at 1:200, Abcam). Positively stained cells were counted to evaluate the host immunological response. SEM was used to examine the luminal surface of the explanted specimens. The degree of tracheal luminal obliteration was calculated by dividing the area of fibrosis by the total area of lumen. The H&E-stained sections were scanned at ×4 magnification, and the area of the obliterated lumen and the total area of lumen were measured using the ImageJ software program (NIH, Bethesda, MD) (Zhao et al., 2013). Histopathologic variables were scored from 0 to 3 to calculate the tracheal necrosis score modified from a method described previously (Iyikesici et al., 2009; Candas et al., 2016) (Supplementary Table S1). Orthotopic explants were dissected latitudinally and longitudinally to examine the anastomosis site stenosis and the viability of the entire explant.

### Statistical analysis

Data were presented as means ± standard deviations. Data were analyzed using one-way analysis of variance (ANOVA) with SigmaStat 4.0 (Systat Software, San Jose, California). *p-*values of less than 0.05 were considered significant.

## Results

### Characterization of DeCTA

**FIGURE 1:**
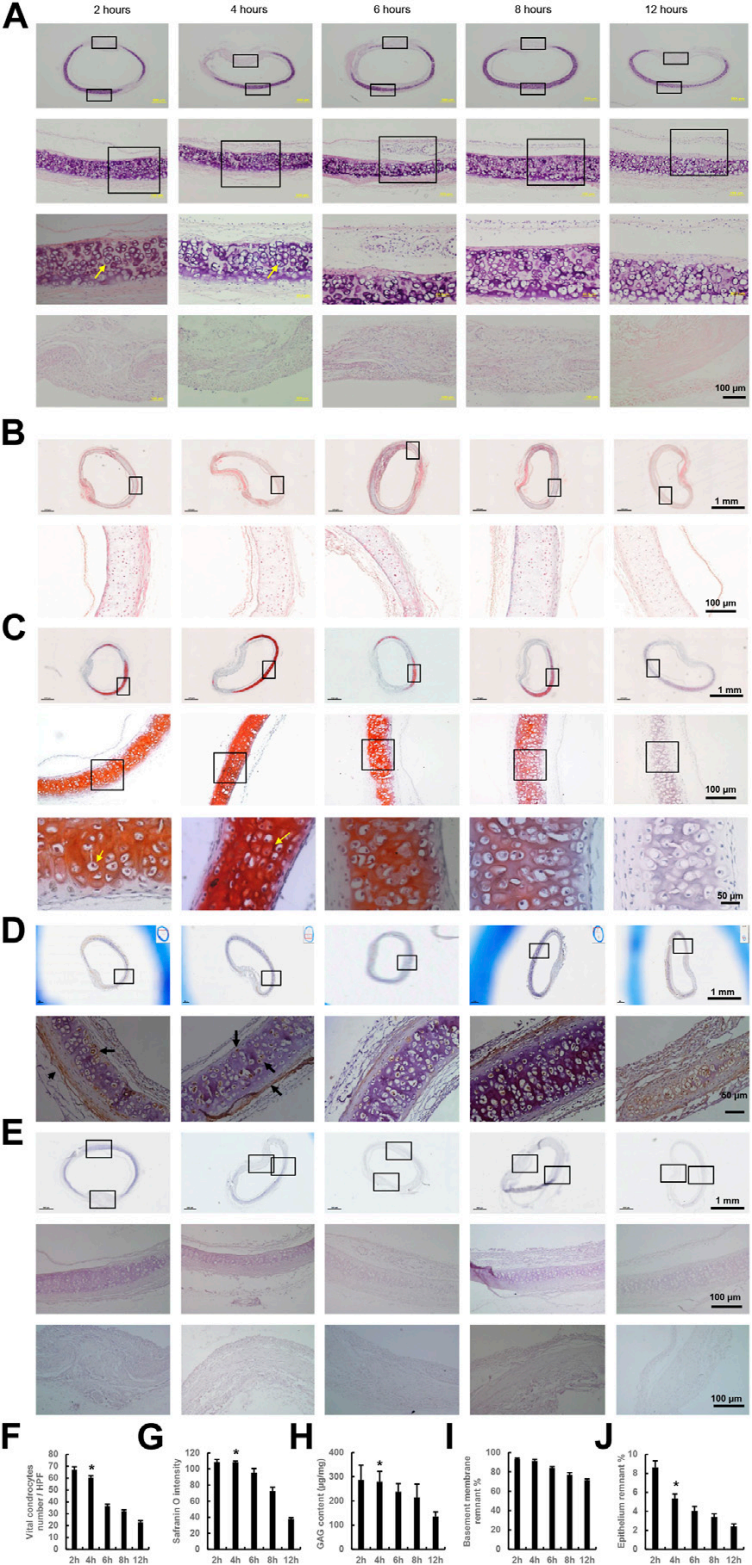
Preparation of DeCTA. **(A)** H&E staining revealed chondrocyte nuclei and lacunar structures in DeCTA after 2, 4, 6, 8, and 12 h of washing with cold PBS; however, the number of normal chondrocytes decreased significantly after 6 h of washing with cold PBS. First (top) row, images of H&E-stained DeCTA. Second row, magnified views of the areas demarcated by the upper black boxes in the first row. Third row, magnified views of the areas demarcated by the black boxes in the second row; the yellow arrows indicate chondrocyte nuclei. Fourth (bottom) row, magnified views of the membrane areas demarcated by the lower black boxes in the first row. **(B,C)** Masson trichrome staining **(B)** and Safranin O staining **(C)** indicated a high density of collagen in the cartilage surrounding the chondrocytes after 4 h of washing with cold PBS; this density gradually diminished with additional washing. In panel **(B)**, the images in the bottom row are magnified views of the areas demarcated by the black boxes in the top row. In panel **(C)**, the images in the bottom row are magnified views of the areas demarcated by the black boxes in the middle row, which themselves are magnified views of the areas demarcated by the black boxes in the top row; yellow arrows indicate chondrocyte nuclei. **(D)** Collagen IV was strongly expressed in basement membrane, lacunar structures and perichondrium after 2 and 4 h of washing with cold PBS, its expression decreased slightly with additional washing. Images in the bottom row are magnified views of the areas demarcated by the black boxes in the top row; black arrows indicate positive staining. **(E)** At all time points, DeCTA showed no OX62 staining, indicating that de-epithelialization completely removed OX62-positive dendritic cells, the most potent antigen-presenting cells within epithelium. Images in the middle and bottom rows are magnified views of the areas demarcated by the black boxes in the top row. **(A–E)** In contrast to the retained chondrocytes, more than 90% of RECs and the submucosa were removed from the tracheal epithelium after 4 h of washing with cold PBS. **(F)** The numbers of normal chondrocytes decreased significantly after 6 h of washing. *p < 0.05 vs. 6, 8 and 12 h. **(G)** The intensity of Safranin O staining decreased significantly after 8 h of washing. *p < 0.05 vs. 8 and 12 h. **(H)** GAG content decreased significantly after 8 h of washing. *p < 0.05 vs. 8 and 12 h. **(I)** The percentage of remnant basement membrane did not differ significantly across time points. **(J)** The percentage of remnant epithelium decreased significantly after 4 h of washing. *p < 0.05 vs. 2 h. ANOVA was used for comparisons among multiple groups, as appropriate. N = 3 biological replicates.

Integrity was preserved in all trachea samples during washing with PBS. H&E staining showed that DeCTA initially had chondrocyte nuclei and lacunar structures, however, normal chondrocyte numbers significantly decreased after 6–12 h of washing in cold PBS (Figures 1A, F). Masson trichrome and Safranin O staining indicated that the density of the extracellular matrix surrounding chondrocytes remained high after 4 h of washing but gradually decreased with additional washing (Figures 1B, C, G). Consistent with the histological analysis, GAG content in the tracheal matrix remained high after 2 and 4 h of washing (Figure 1H). Tracheal basement membrane was also well preserved after 2 and 4 h of washing (Figures 1A–C, I). Collagen IV, the major component of basement membrane, was strongly expressed in basement membrane and lacunar structures after 2 and 4 h of washing but gradually decreased thereafter (Figure 1D). The absence of OX62 staining indicated that dendritic cells, the most potent antigen-presenting cells within epithelium, were completely removed during de-epithelialization (Figure 1E). In contrast to its effect on chondrocytes, a 4-h wash with cold PBS removed more than 90% of epithelial cells and the submucosa from the trachea samples, while the left epithelium (∼5.35% ± 0.49%) was lacking normal structure (Figures 1A–C, J). These results suggested that a 4-h wash with cold PBS sufficiently removed epithelium from CTA but did not remove its chondrocytes, extracellular matrix content, and tracheal rigidity. Therefore, the DeCTA that resulted from the 4-h wash with PBS was selected for ensuing experiments.

**FIGURE 2:**
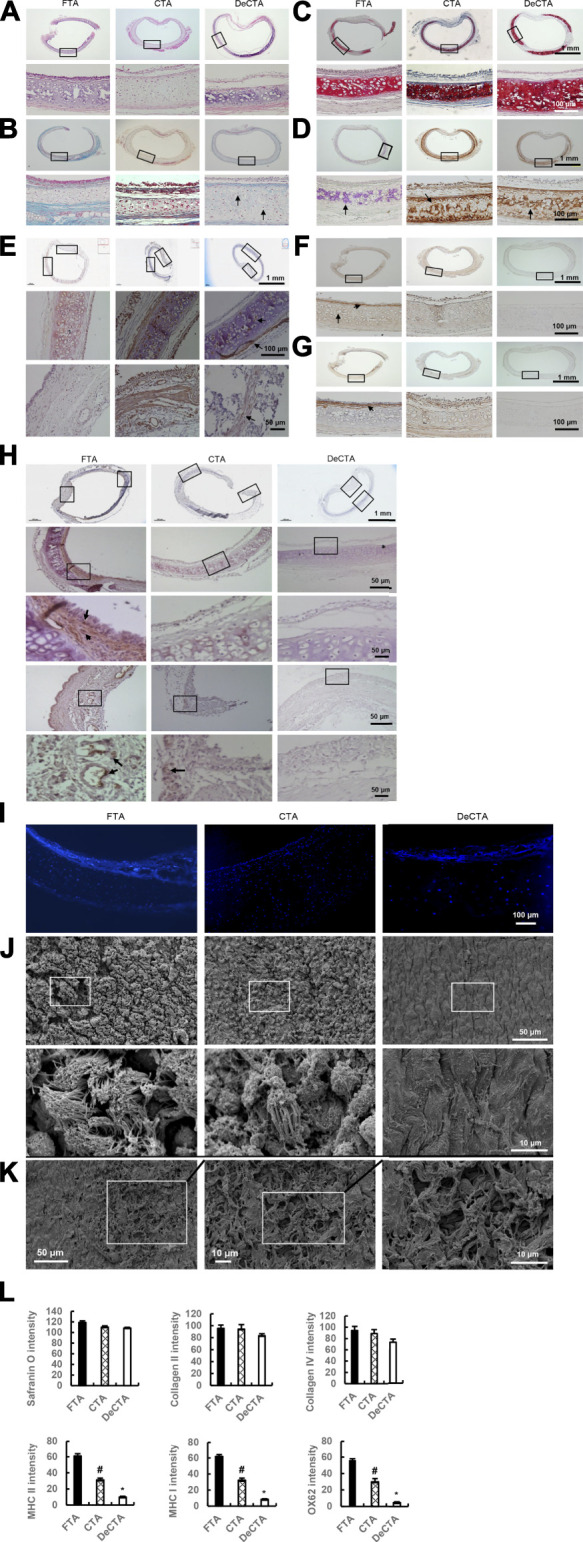
Characterization of DeCTA. **(A)** H&E staining showed that chondrocytes were well preserved, most of the epithelium was removed, and the basement membrane was maintained in DeCTA. Compared with that in FTA or CTA, cartilage in DeCTA had some slight calcification, but this difference was not significant. Most of the epithelium in CTA was preserved. **(B)** Masson trichrome staining showed that DeCTA had numerous normal chondrocytes with clear nuclei in lacunar structures, which were surrounded by dense collagen (blue). The chondrocytes in FTA, CTA, and DeCTA had similar features. Black arrows indicate chondrocyte nuclei. **(C,D)** Safranin O staining **(C)** and collagen II staining **(D)** showed that FTA, CTA, and DeCTA all had cartilage with a dense collagen matrix. The images in the bottom rows of **(A–D)** are magnified views of the areas demarcated by the black boxes in the top rows of those panels. **(E)** Collagen IV, an important component of basement membrane, was distributed in the basement membrane, lacunar structures and perichondrium in FTA, CTA, and DeCTA. The images in the middle and bottom rows are magnified views of the areas demarcated by the black boxes in the top row; black arrows indicate positive staining of collagen IV. **(F–H)** Compared with that in FTA, MHC I **(F)**, MHC II **(G)**, and OX62 **(H)** staining were significantly decreased in CTA and even absent from DeCTA. OX62-positive dendritic cells (black arrows), which are important antigen-presenting cells, were distributed in the subepithelial layer around vascular structures in FTA. In panels **(F,G)**, the images in the bottom rows are magnified views of the areas demarcated by the black boxes in the top rows. In **(H)**, the images in the third (middle) and fifth (bottom) rows are magnified views of the areas demarcated by the black boxes in the second and fourth rows, respectively, which are themselves magnified views of the areas demarcated by the black boxes in the first (top) row. Black arrows indicate positive staining. **(I)** DAPI staining showed that chondrocytes were present in FTA, CTA, and DeCTA. **(J)** SEM analysis confirmed that the epithelium was removed and the basement membrane remained intact in DeCTA. The images in the bottom row are magnified views of the areas demarcated by the black boxes in the top row. **(K)** SEM revealed several areas of basement membrane pinpoint defects, which resulted in the exposure of underlying collagen fibrils, after 6 h of washing with cold PBS. The average size of these areas of destruction was about 0.01 mm^2^. **(L)** Safranin O, collagen II, and collagen IV staining intensity did not differ significantly among FTA, CTA, and DeCTA. Compared with FTA, both CTA and DeCTA had significantly diminished MHC I, MHC II, and OX62 staining intensity. ^#^ *p* < 0.05 vs. FTA; *p < 0.01 vs. FTA and CTA. ANOVA was used for comparisons among multiple groups, as appropriate. N = 3 biological replicates.

To further characterize DeCTA, we compared it with FTA and CTA. H&E, Masson trichrome, and DAPI staining revealed high percentage of viable chondrocytes were preserved in both CTA (∼84.3 ± 3.4% normal chondrocytes ratio of CTA/FTA) and DeCTA (∼61.2 ± 2.9% normal chondrocytes ratio of CTA/FTA) as well as in FTA (Figures 2A, B, I). Safranin O, collagen II, and collagen IV staining intensity did not differ significantly among FTA, CTA, and DeCTA (Figures 2C–E, L). Compared with that in FTA, staining for MHC I, MHC II, and OX62 were significantly decreased in CTA and even absent in DeCTA (Figures 2F–H, L). SEM analysis confirmed that the epithelium was removed, whereas the basement membrane remained intact, in DeCTA (Figure 2J). However, a 6-h wash with PBS resulted in partial basement membrane destruction with exposure of the underlying collagen fibrils (Figure 2K). SEM analysis revealed pinpoint defects in the basement membrane that were undetectable with regular histological analysis.

**FIGURE 3:**
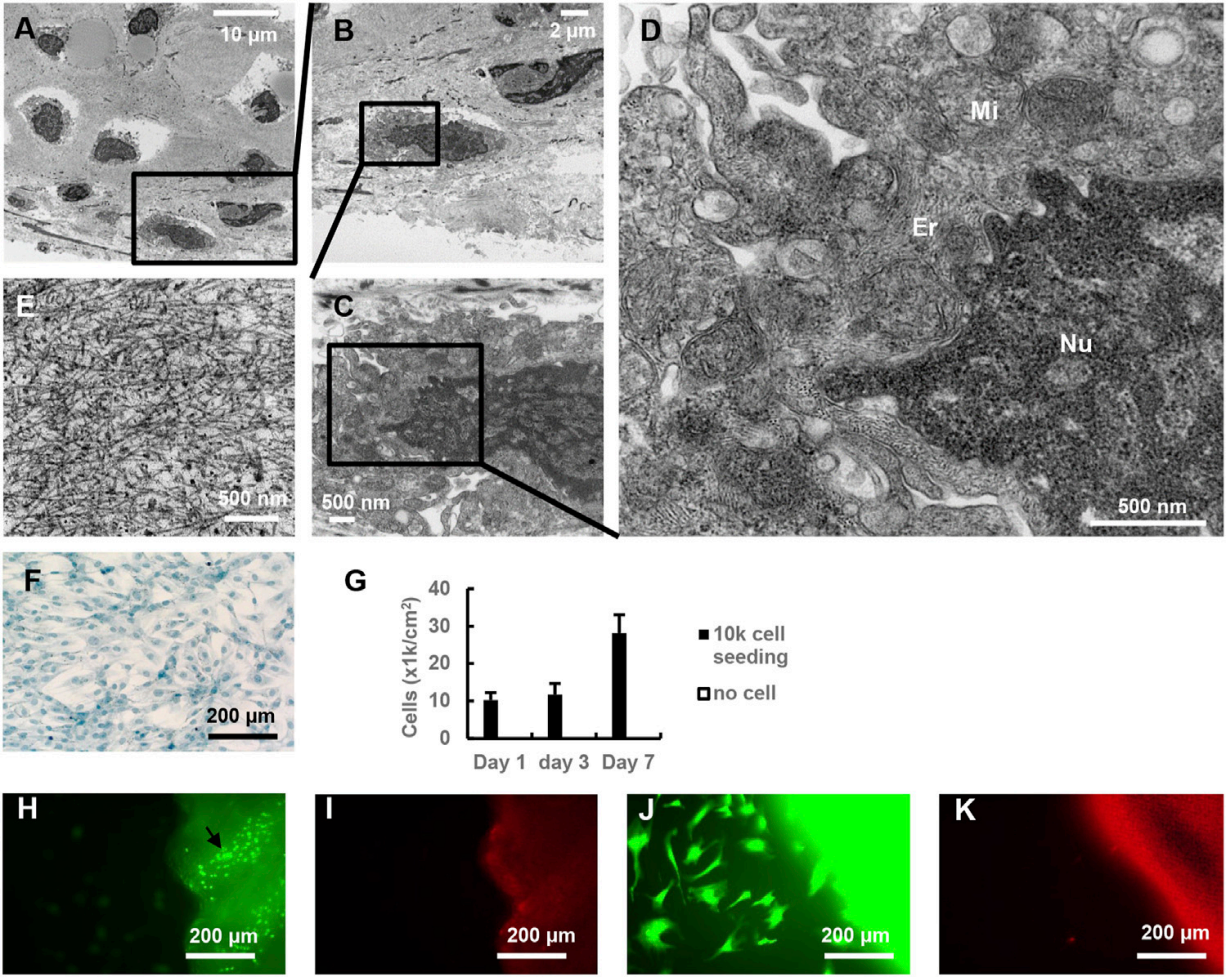
Characterization of chondrocytes in DeCTA. **(A–D)** TEM showed supermicroscopic structures of normal chondrocytes in DeCTA. Mi, mitochondria; Er, endoplasmic reticulum; Nu, nucleus. **(E)** Dense extracellular matrix surrounding chondrocytes in lacunae. **(F)** Alcian blue–stained chondrocytes isolated from DeCTA and cultured at day 3. **(G)** MTT assay showed chondrocyte proliferation from day 1–7. **(H)** Live/dead staining showed living chondrocytes (black arrows) in DeCTA. Few vital epithelium cells were detected. Calcein AM staining appears as green fluorescence. **(I)** Live/dead staining showed few dead cells in DeCTA. EthD-1 staining appears as red fluorescence. **(J)** Live/dead staining showed living BM-MSCs seeded on DeCTA. **(K)** No dead BM-MSCs were detected in DeCTA. N = 3 biological replicates.

Chondrocyte was further evaluated using TEM analysis. Chondrocytes in the cartilage of DeCTA had clear supermicroscopic structures, including nuclei, mitochondria, and endoplasmic reticulum (Figures 3A–E). These normal structures are vital to cellular biological functionality. Coinciding with this finding, MTT assay revealed that chondrocytes isolated from DeCTA were able to proliferate (Figures 3F, G). Live/dead staining of BM-MSCs cocultured with DeCTA demonstrated not only viable chondrocytes in cartilage but also healthy BM-MSCs surrounding cartilage, which suggests that DeCTA is non-toxic and has histocompatibility. Particularly, very few viable epithelium cells were detected in DeCTA (Figures 3H–K).

### Pre-epithelialization of DeCTA

**FIGURE 4:**
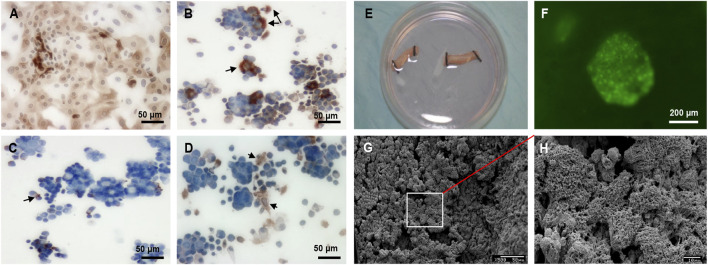
REC culture and seeding in DeCTA. **(A)** RECs were positive for cytokeratin peptide 17. **(B)** Beta tubulin IV–positive ciliated columnar cells. Black arrows indicate cilia. **(C)** Black arrows indicate P63-positive basal cells. **(D)** Black arrows indicate mucin 5AC–positive goblet cells. **(E)** RECs were seeded into DeCTA. **(F)** Live/dead staining showed clusters of healthy RECs adhered to DeCTA. **(G)** SEM revealed that RECs adhered to and re-epithelialized DeCTA with 1 day of culturing. **(H)** Magnified view of the area demarcated by the white box in panel **(G)** N = 3 biological replicates.

RECs were successfully cultured and then identified with immunohistochemical staining. Most RECs were positive for cytokeratin peptide 17 at passage 0 (Figure 4A). RECs at passage 0 included beta tubulin IV–positive ciliated columnar cells (Figure 4B), P63-positive basal cells (Figure 4C), and mucin 5AC–positive goblet cells (Figure 4D). Healthy ciliated columnar cells functioned normally to move cell clusters (Supplementary Video S1). Live/dead staining revealed that RECs adhered to DeCTA well (Figures 4E, F). SEM showed that DeCTA cocultured with RECs was successfully epithelialized in the luminal surface (∼81.3 ± 5.6%), yielding ReCTA (Figures 4G, H).

### *In vivo* assessment of heterotopically implanted ReCTA

**FIGURE 5:**
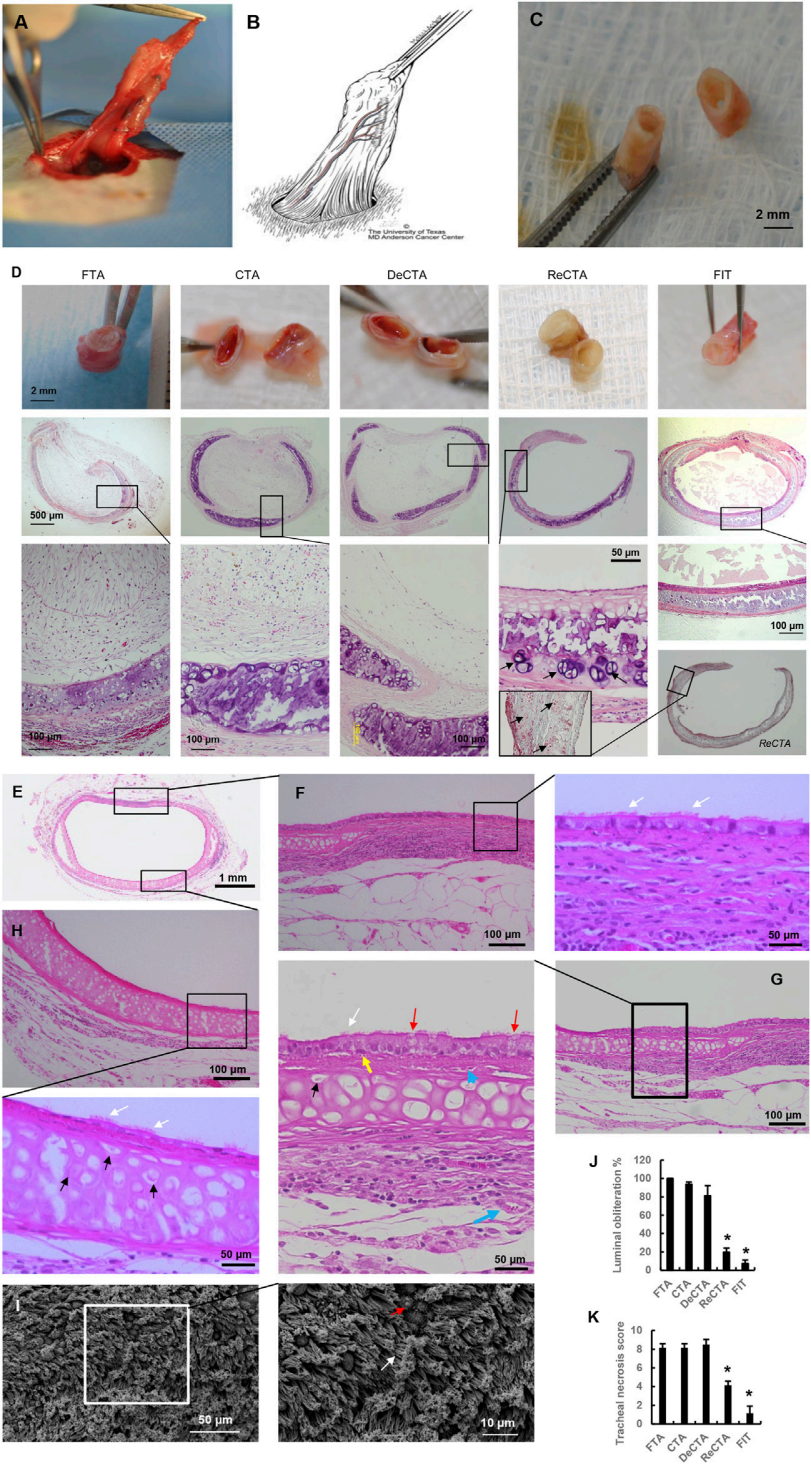
*In vivo* assessment of heterotopically implanted ReCTA. **(A)** ReCTA was neovascularized by a pedicled adipose tissue flap subcutaneously in the groin area 30 days postoperatively. **(B)** Scheme of the pedicled adipose tissue flap based on the superficial epigastric blood vessels in the groin area. Its micro-branches anastomosed with ReCTA to provide the blood supply. **(C)** ReCTA retained luminal patency 30 days postoperatively. **(D)** Histological analysis showed that fibrosis obliterated FTA, CTA, and DeCTA. There was calcification necrosis in the center of the cartilage. Unlike FTA, CTA, and DeCTA, both ReCTA and FIT retained luminal patency and structural integrity with complete epithelial cell coverage. There was some calcification necrosis in the center of the cartilage in ReCTA; however, lots of vital chondrocytes (black arrows) were in the cartilage, especially in the periphery. **(E–I)** Histological features of explanted ReCTA 30 days postoperatively. Both the membrane part **(F,G)** and cartilage part **(H)** of ReCTA were completely epithelialized by RECs, which included cilia cells (white arrows), basal cells (yellow arrows), and goblet cells (red arrows). Microvascular blood vessels (blue arrows) grew in both the subepithelium and adventitia. Vital chondrocytes with clear nuclei (black arrows) were distributed in the cartilage, especially in the periphery. SEM revealed healthy cilia (white arrows) and goblet cells (red arrows) in the epithelium **(I)**. **(J)** Compared with FTA, CTA, and DeCTA, both ReCTA and FIT had significantly lower percentages of luminal obliteration. *p < 0.05 vs. FTA, CTA, and DeCTA. **(K)** Compared with FTA, CTA, and DeCTA, both ReCTA and FIT had significantly lower tracheal graft necrosis scores. *p < 0.05 vs. FTA, CTA, and DeCTA. ANOVA was used for comparisons among multiple groups, as appropriate.

ReCTA maintained normal rigidity with some slight contracting 30 days postoperatively. It was integrated with and vascularized by the surrounding adipose tissue flap (Figures 5A, B). Under surgical microscope observation, its lumen was patent, and the neo-trachea had preserved elasticity and strength to resist compression (Figure 5C; Supplementary Video S2). In contrast, FTA, CTA, and DeCTA were obliterated and severely contracted, resulting in deformity (Figure 5D). Histological analysis confirmed that ReCTA maintained normal structural integrity and had epithelium, cartilage, and luminal patency. In all grafts, the periphery of the cartilage had a high percentage of viable chondrocytes (∼31.8% ± 9.5%), indicating that cartilage maximally evaded host immune recognition (Figure 5D). FTA, CTA, and DeCTA were almost completely obliterated by lamina propria hyperplasia, which was characterized by epithelium loss in FTA and CTA and abundant fibroblasts in FTA, CTA, and DeCTA, while only varied subepithelium thickness was observed in ReCTA (∼70.5 ± 15 µm). H&E staining and SEM analysis revealed cilia in the ReCTA epithelium, especially in the posterior wall of tracheal membrane fascia area, where the blood supply was easily established earlier than in other parts (Figures 5E–I). Besides ciliated cells, both basal cells and goblet cells, which are responsible for epithelial proliferation and mucus secretion, respectively, were distributed along the epithelium (Figures 5E–I). In FIT, the entire lining was covered by normal pseudostratified columnar epithelium with cilia, whereas in ReCTA the entire lining was covered by a monolayer of epithelial cells, of which only about 15.0% ± 2.3% had cilia. Partial cartilage necrosis and calcification were present in the centers of all grafts, but compared with FTA, CTA, and DeCTA, both ReCTA and FIT had significantly lower luminal obliteration ratios and tracheal necrosis scores (Figures 5J, K).

**FIGURE 6:**
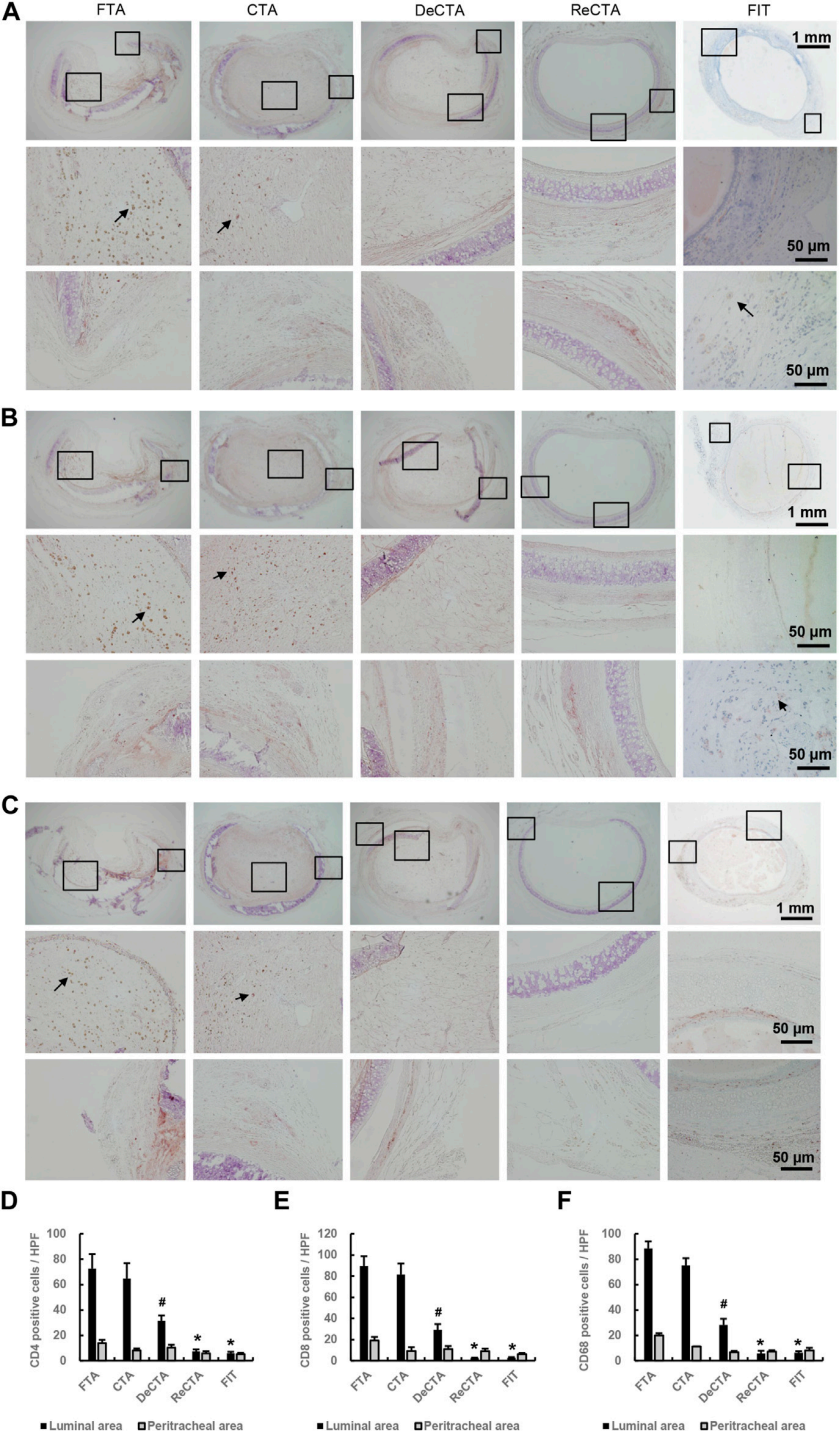
Infiltration of inflammatory cells in tracheal allografts. **(A–C)** Immunohistochemical staining revealed CD4^+^ T cells **(A)**, CD8^+^ T cells **(B)**, and CD68^+^ macrophages **(C)** infiltrating the lumens of FTA and CTA but detected few of these immune cells in DeCTA, ReCTA, or FIT. In panels **(A–C)**, images in the middle (showing lumen) and bottom rows (showing cartilage and adventitia) are magnified views of the areas demarcated by the black boxes in the top rows; black arrows indicate the infiltrating cells. **(D–F)** Compared with FTA and CTA, DeCTA, ReCTA, and FIT had significantly fewer CD4^+^ cells **(D)**, CD8^+^ T cells **(E)**, and CD68^+^ macrophages **(F)** infiltrating their luminal and peritracheal areas. ^#^ *p* < 0.05 vs. FTA, CTA; *p < 0.01 vs. FTA, CTA. ANOVA was used for comparisons among multiple groups, as appropriate.

Immunohistochemistry revealed significant immunorejection in FTA and CTA (Figures 6A–C). Compared with DeCTA, ReCTA, and FIT, both FTA and CTA had dramatically more CD4^+^ T-cell, CD8^+^ T-cell, and CD68^+^ macrophage infiltration in the lumen and adventitial areas (Figures 6D–F), indicating that the rejection was initiated by the epithelium. Antigen-presenting cells, such as macrophages and dendritic cells, could have presented the alloantigen to T cells, activating them. The consequent molecular signaling cascade resulted in severe inflammation and cellular proliferation, which further caused graft obliteration and loss of function (Figure 6). Compared with FTA and CTA, DeCTA had less T cell and macrophage infiltration, suggesting that its obliteration was mainly initiated by wound healing and fibrosis rather than immunorejection (Figure 6). The numbers of infiltrating CD4^+^, CD8^+^, and CD68^+^ cells in ReCTA and FIT did not differ significantly, indicating that ReCTA did not stimulate immunorejection (Figure 6).

### *In vivo* assessment of orthotopically implanted DeCTA

**FIGURE 7:**
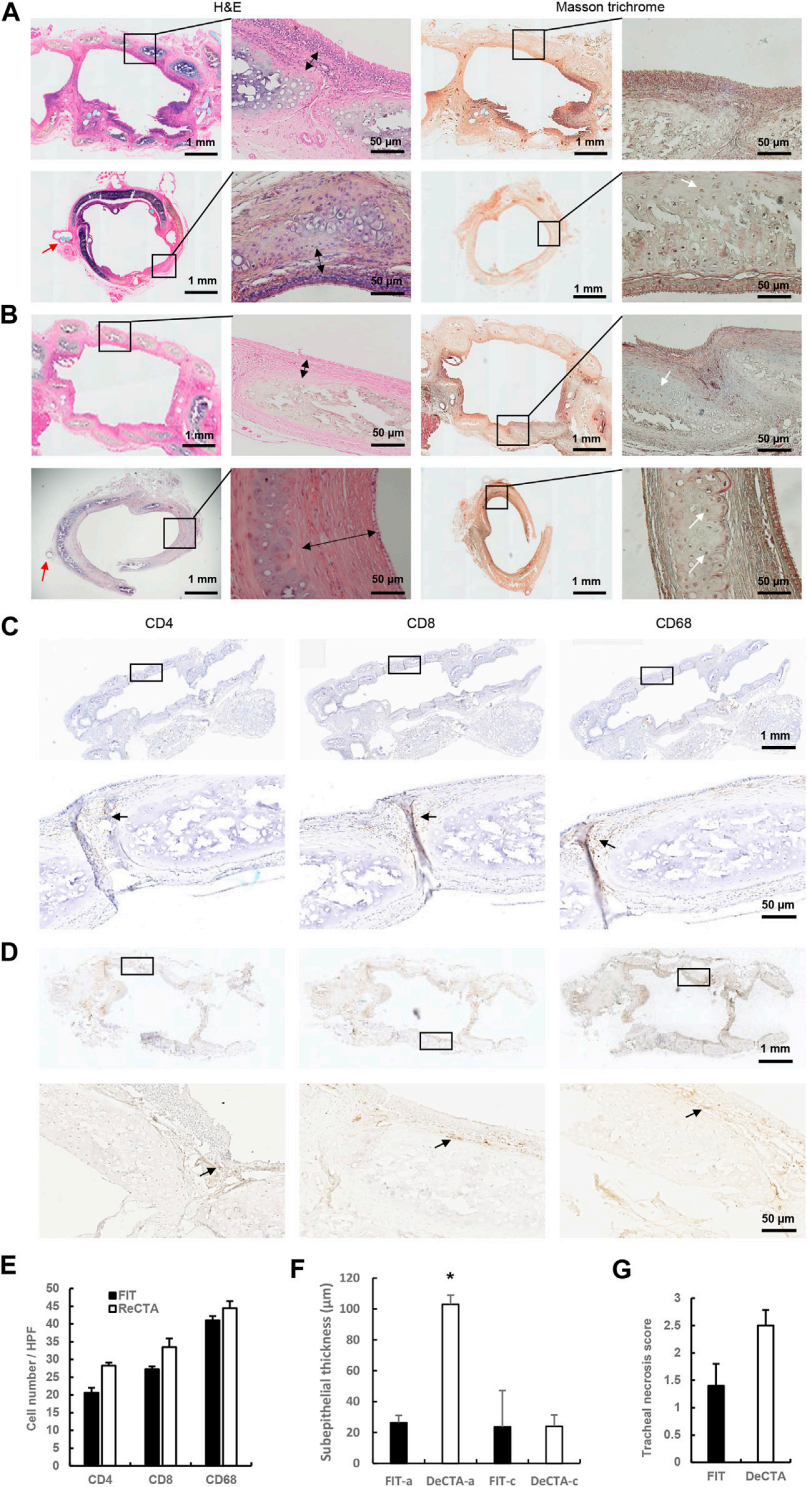
*In vivo* assessment of orthotopically implanted DeCTA 3 months postoperatively. **(A,B)** H&E and Masson trichrome staining showed that both FIT **(A)** and DeCTA **(B)** retained luminal patency with complete structural integrity and strong mechanic features and were completely covered by epithelial cells. Top row, images of longitudinal sections; bottom row, images of cross sections of the anastomosis sites. White arrows indicate vital chondrocytes; red arrows indicate surgical sutures; double-headed arrows indicate subepithelial thicknesses. **(C,D)** Both FIT **(C)** and DeCTA **(D)** showed infiltration of a few CD4^+^ T cells, CD8^+^ T cells, and CD68^+^ macrophages (black arrows) in the subepithelial area. Images in the bottom rows show magnified views of the areas demarcated by the black boxes in the top rows. **(E–G)** The necrosis scores **(G)** and inflammatory cell numbers **(E)** for DeCTA and FIT did not differ significantly; however, DeCTA had significantly higher subepithelial thickness at the anastomosis site **(F)**. *p < 0.05 vs. anastomosis site of FIT. FIT-a, anastomosis site of FIT; DeCTA-a, anastomosis site of DeCTA; FIT-c, graft center of FIT; DeCTA-c, graft center of DeCTA. ANOVA was used for comparisons among multiple groups, as appropriate.

To evaluate if the cartilage rigidity of DeCTA can endure tracheal movement *in vivo*, we compared orthotopic implants of DeCTA and FIT, which were placed in 5 rats each. One rat implanted with DeCTA died 9 days postoperatively because of airway obstruction and collapse at the trachea anastomosis site, but the remaining 4 rats implanted with DeCTA survived until they were humanely killed 3 months postoperatively. Explanted DeCTA showed luminal patency as well as rigidity with elasticity and strength. Histological analysis showed that, like FIT, DeCTA had a normal tracheal structure. As in the heterotopic implantation model, partial cartilage calcification necrosis occurred in the centers of the grafts. However, there was a considerable percentage of viable chondrocytes (∼32.1% ± 6.5%) distributed in the periphery, and these cells sufficiently supported the trachea’s structural integrity and functionality, enabling its adaptation to neck movement. Unlike in the heterotopic implantation model, the tracheal lining had a high percentage of cilia cells (∼86.0% ± 8.6%), indicating that it was completely covered by the cells and that host epithelial cells successfully migrated from the anastomosis site and proliferated to cover the grafts (Figures 7A, B). In both FIT and DeCTA, the anastomosis area had CD68^+^ macrophages, CD4^+^ T cells, and CD8^+^ T cells (Figures 7C, D), albeit in significantly smaller numbers than those in FTA and CTA in the heterotopic implantation model (Figures 7C, D). The numbers of these cells in DeCTA and FIT in the orthotopic implantation model did not differ significantly (Figure 7E). The subepithelium at the anastomosis site was thicker in DeCTA than in FIT; however, the subepithelial thicknesses at the centers of the grafts did not differ significantly (Figures 7A, B, F). Subepithelial thickness did not affect luminal patency during the 3-month observation period, and the tracheal necrosis scores for DeCTA and FIT did not differ significantly (Figure 7G).

## Discussion

The goal of tracheal reconstruction is to provide a non-collapsible airway with a stable epithelial lining and reliable, well-vascularized tissue coverage (Yu et al., 2006; Delaere et al., 2010; Yu et al., 2011; Rendina and Patterson, 2022; Yang et al., 2023). To achieve this goal, in the present study, we applied a partially decellularized tracheal scaffold to provide airway support and successfully covered its lumen with RECs to provide a functional respiratory lining. Our findings demonstrate that heterotopically implanted engineered neo-tracheas can be successfully neovascularized with an adipo-fascial flap and that orthotopically implanted neo-tracheas can have long-term survival with full function. This proof-of-concept study provides a new strategy for tracheal tissue engineering and could be clinically translated to reconstruct longer tracheal defects.

Completely removing epithelium from tracheal allografts while retaining an intact basement membrane and viable cartilage presents a challenge. Liu et al. (2000) recently reevaluated their previous studies over the past 2 decades and found that chemical detergent could effectively remove most of the epithelium and mixed glands from dog trachea. Compared with fresh tracheal cartilage, the de-epithelialized tracheal cartilage had significantly less GAG and collagen II, but the viable chondrocyte ratio was about 50%, and in a dog orthotopic transplantation model, the remaining chondrocytes played key roles in preserving the patency and rigidity of the resultant allograft and preventing the development of granulation tissue during the initial transplantation period, thereby supporting the graft’s long-term survival (Lu et al., 2018). The authors concluded that maintaining cartilage viability is necessary for successful immunosuppressant-free allotransplantation; however, the chemical detergent inevitably damaged chondrocytes during de-epithelialization (Liu L et al., 2002). Liu et al. (2021) and Liu et al. (2022) also reported that a modified detergent-based decellularization protocol eliminated all cell populations except chondrocytes in mouse trachea. Although the number of vital chondrocytes, which was initially small (∼2.00% ± 3.46%), increased after implantation (∼25.97% ± 10.99%), chondrocyte viability was still poor overall, and this may have been associated with the higher mortality rate of the host animals (∼58%). Hence, chondrocyte viability plays a critical role in maintaining the biomechanical properties necessary to prevent stenosis or collapse in tracheal replacement (Liu et al., 2021; Liu Y et al., 2022). To protect tracheal chondrocytes in their study, Aoki et al. used a brief (3-h) detergent-based treatment with sodium dodecyl sulfate to remove only the epithelium and maintain the structural integrity of porcine tracheal grafts while keeping most of the cartilage alive *in vitro* (Aoki et al., 2019). In another series of studies, Hysi et al., 2014 heterotopically and orthotopically transplanted epithelium-denuded CTA into rabbit and porcine models. Rather than using a chemical-based method, they successfully applied a mechanical/surgical-based approach to remove the tracheal mucosa and obtain an epithelium-denuded tracheal allograft with preserved viable cartilage (Hysi et al., 2014; Hysi et al., 2015; De Wolf et al., 2017). Our findings are consistent with these previous reports. We found that the rat tracheal epithelium with most of its antigenicity could be easily removed after cryopreservation; even simply washing the trachea with cold PBS could remove most of the epithelium. Our SEM and histological analyses showed that most of the basement membrane was maintained, which is key to re-epithelialization (Delaere and Van Raemdonck, 2014), and our live/dead cell assay and histological and TEM analyses showed that cartilage viability was maintained, as evidenced by the high percentage of viable chondrocytes (∼61.2% ± 2.9%). Our *in vivo* studies confirmed that the resultant DeCTA had the biomechanical properties the graft required to resist compression and remain patent. However, vital chondrocytes were predominantly distributed around the periphery of DeCTA, which indicated that cryoprotectants did not permeate the tracheal graft entirely, especially at its center. Although the remaining vital chondrocytes and surrounding dense extracellular matrix sufficiently maintained the tracheal structural integrity both *in vitro* and *in vivo*, additional studies are warranted to develop novel permeating and non-permeating cryoprotectants and to improve both the cryopreservation program [for example, by using vitrification cryopreservation (Yong et al., 2020)] and the partial de-epithelialization procedure to maximize the viability of tracheal cartilage (Sotres-Vega et al., 2006; Nakanishi, 2009; Zang et al., 2011; Delaere and Van Raemdonck, 2014).

Cryopreservation not only enables the long-term storage of viable allografts but also induces an immunomodulatory effect to improve allograft survival. Studies have shown that both the reduced antigenicity of the trachea (i.e., that conveyed by MHC I and II) that results from the partially denuded epithelium and the degeneration of chondrocytes by cryopreservation contribute to the immunomodulatory effect (Nakanishi, 2009). In other studies, cartilage sufficiently maintained tracheal rigidity in orthotopically implanted CTA (Sotres-Vega et al., 2006; Nakanishi, 2009), whereas the remaining epithelium (10%–40%) was rejected and gradually replaced by recipient epithelium (Mukaida et al., 1998; Genden et al., 2003). These results suggest that successful CTA implantation depends in part on the immune privilege of the cartilage. The experimental use of CTA for shorter defects has shown some success, but its use for longer defects has been largely unsuccessful (Nakanishi, 2009), primarily because the host epithelium cannot proliferate rapidly enough to entirely cover the exposed graft and prevent its fibrosis, stenosis, infection, and/or collapse (Hysi et al., 2015). This occurred in the heterotopic implantation experiments in the present study. Although CTA had significantly reduced antigenicity, the remaining epithelium still stimulated immunorejection, as evidenced by the high numbers of CD4^+^ T cells, CD8^+^ T cells, and CD68^+^ macrophages infiltrating the lumens of the implants. In contrast, in DeCTA, the complete removal of the epithelium resulted in significantly fewer of those immune cells in the lumen; however, the lumen was still completely obliterated by fibrosis. Therefore, the successful transplantation of CTA and DeCTA requires appropriate epithelium cover to prevent immunorejection and lamina propria hyperplasia and ensure functional recovery (Delaere et al., 2010; Delaere and Van Raemdonck, 2014; Hysi et al., 2015). To further delineate the characterization of the immunological response on CTA and DeCTA, more details of macrophages subtypes infiltration (e.g., M1 and M2 macrophages) and their functionality need to be investigated in the next step.

A bioengineering approach could significantly enhance the pre-epithelialization of the tracheal bioscaffold, but the outcomes of such an approach depend on the cell types and seeding procedure used. In the first successful clinical use of tracheal allotransplantation, buccal mucosa was used to cover the fresh tracheal allograft (Delaere et al., 2010). However, the slow repopulation of the tracheal lining by grafted recipient oral mucosa cells significantly hinders the wide application of this therapy, and mucosa by itself cannot replace the full function of real RECs (Delaere et al., 2010; Delaere et al., 2012). Therefore, researchers have turned to a tissue-engineering approach to pre-epithelialize tracheal grafts with RECs (Aoki et al., 2019). However, the use of RECs to prepopulate de-epithelialized tracheal allograft or DeCTA for transplantation has not been reported (Liu et al., 2000; Hysi et al., 2014; Hysi et al., 2015; De Wolf et al., 2017; Lu et al., 2018). In the present study, we successfully isolated RECs from syngeneic donor rats and cultured them. The cultured RECs included cilia cells, basal cells, and goblet cells, all of which play important roles in effecting the full functional recovery of the neo-trachea. RECs seeded in the DeCTA lumen *in vitro* adhered and grew because the basement membrane was intact after de-epithelialization. Complete preservation of the basement membrane is important to epithelium healing because RECs cannot grow properly if the basement membrane is damaged (Delaere and Van Raemdonck, 2014). Without the coverage of the RECs, fibroblast hyperplasia would occur, resulting in subepithelial lamina propria tissue thickening and lethal stenosis (Hysi et al., 2015; Liu Y et al., 2022). In the heterotopic implantation model in the present study, RECs completely covered the lumens of explanted ReCTA, and these grafts had not only tracheal patency (with the smallest obliteration ratio among all grafts) but also functional epithelial cells. Moreover, SEM and histological analyses revealed healthy cilia cells growing in the epithelium. It is well known that cilia are crucial to the removal of sputum to prevent and reduce infection. Histological analysis showed that more cilia cells grew in the posterior wall of the tracheal membrane area than in the cartilage area, possibly because vascularization occurred more easily in the membrane area, which has only soft tissue. Although cilia, basal, and goblet cells were all maintained in ReCTA, in most areas, they constituted only a monolayer epithelium, which was much thinner than the pseudostratified columnar epithelium in FIT. Contributing to this may have been the static culture system we used for the pre-epithelialization of ReCTA. To improve epithelialization, researchers must develop and optimize more comprehensive, cutting-edge culturing strategies; for example, dynamic organ culturing using a bioreactor could be employed to enhance tracheal scaffold epithelialization (Aoki et al., 2019). However, this would require achieving a delicate balance between the duration of the *in vitro* culturing of RECs on DeCTA and the duration of cartilage ischemia to avoid excessive chondrocyte apoptosis. An ideal strategy for achieving this goal could be an *in vivo* engineering approach in which heterotopic vascularization is simultaneously incorporated with the induction of REC proliferation and maturation on DeCTA after its initial adhesion *in vitro*.

In previous studies, de-epithelialized tracheal allograft showed a significant loss of chondrocyte viability after implantation in both long-term dog and short-term rabbit orthotopic implantation models (Hysi et al., 2015; Lu et al., 2018). In the present study, we also found that chondrocyte viability decreased after both short-term heterotopic and long-term orthotopic implantation. As Tanaka et al. (2003) reported, this reduced chondrocyte viability may be closely related to the fact that the relative ischemia of the cartilage during the initial revascularization phase could provoke apoptosis and subsequent dystrophic cartilage calcification. Because the tracheal graft is prone to ischemia, the neovascularization of the neo-trachea must be established as early as possible. Researchers have attempted numerous strategies to enhance the neovascularization of tracheal implants. Because the trachea lacks a dominant vasculature, it cannot be transferred with microvascular anastomosis; rather, its blood supply must be derived secondarily from wrapping flaps. Local pedicled flaps [e.g., sternohyoid muscle flaps (Luo et al., 2013)] and distal pedicled flaps [e.g., omentum flaps (Masaoka et al., 2002)] have been used to wrap tracheal grafts and constructs to form neo-trachea flap to provide a blood supply. In orthotopic models, such flaps are fabricated either immediately or in 2 stages; in the latter case, they are first prefabricated in a heterotypical position and then transferred to the orthotopic position in the neck (Sykes, 2010). Because neck and/or tracheal movement can dramatically affect the anastomosis between the vascular branch of the flap and the tracheal graft, 2-stage fabrication, such as that used in the present study, is most commonly used. With its pedicle of superficial epigastric arteries and veins, the neo-trachea flap fabricated in the groin position can be transferred to the neck as a vascularized composite free flap. Because the rat model we used allowed the orthotopic transfer of only a short tracheal graft, whose survival could be achieved through its vascularization with the adjacent recipient trachea, we did not perform a 2-stage transfer in the orthotopic model in the present study. The presence of viable epithelial cells and chondrocytes in neo-trachea indicated that neovascularization occurred shortly after heterotopic and orthotopic implantation. Besides flaps, angiogenic growth factors, such as VEGF or bFGF, have been applied directly to tracheal grafts and constructs to improve neovascularization (Sung and Won, 2001; Nakamura et al., 2019). Other approaches, such as those employing MSCs, have been used to enhance epithelium regeneration and graft vascularization (Han et al., 2011). An “all-in-one” approach incorporating the abovementioned methods could be used to promote earlier neovascularization in tracheal constructs in further studies.

In the present study, one of the rats with orthotopically implanted DeCTA died postoperatively from airway obstruction and collapse, indicating that the approach needs to be refined to further improve cartilage vitality and tracheal integrity. In the rats with orthotopic implants, the thickened subepithelium at the anastomosis site may have been caused by surgical injury and the exposure of the lining in the early postoperative stages, which may have stimulated cellular hyperplasia. However, in terms of graft epithelialization, the mechanical strength of the cartilage, luminal patency, and the overall survival rates of the animals, our results are promising as those of previous studies of orthotopically implanted tracheal allografts in rodent models (Candas et al., 2016; Liu et al., 2021). Our heterotopic implantation studies demonstrated the successful pre-epithelialization and neovascularization of the neo-trachea, whereas our orthotopic implantation studies demonstrated that the cartilage of DeCTA had sufficient biomechanical strength to bear neck movement and compression to ensure normal airway function. These findings provide a proof-of-concept for applying ReCTA for tracheal tissue engineering. These findings also demonstrate that the implantation of small grafts of ReCTA presents few technical challenges, which should enable us to rapidly and efficiently advance our technique to testing in large animal models, in which we will ultimately use 2-stage transplantation to reconstruct longer tracheal defects.

## Conclusion

DeCTA effectively had no epithelial allo-antigenicity and had a well-maintained 3-dimensional architecture, an intact basement membrane, vital chondrocytes, and cartilage with structural integrity and strong mechanical properties. DeCTA was also non-cytotoxic, having no side effects on RECs’ morphology or adhesion, indicating that it has potential as a bioscaffold for tracheal tissue engineering by providing a mimetic niche *in vivo*. Our findings in heterotopic and orthotopic implantation models confirmed that the tracheal cartilage of ReCTA has immune privilege and has sufficient mechanical properties to bear neck movement and compression; indicated that the pre-epithelialization of DeCTA, resulting in ReCTA can prevent fibrosis obliteration and maintain lumen/airway patency in neo-trachea; and showed that a pedicled adipose tissue flap can be easily integrated with ReCTA to achieve neovascularization. In conclusion, a 2-stage bioengineering approach can be used to create and prevascularize ReCTA to form neo-trachea flap, whose intact basement membrane and viable chondrocytes contribute to its structural integrity, thus providing a promising tracheal reconstruction strategy that warrants further testing in large animal models. Ultimately, ReCTA could be translated to the clinic for the reconstruction of large tracheal defects.

## Data Availability

The original contributions presented in the study are included in the article/Supplementary Material, further inquiries can be directed to the corresponding authors.

## Supplementary material

SUPPLEMENTARY VIDEO S1 Isolation of RECs. Cilia drive the movement of healthy cell clusters.

SUPPLEMENTARY VIDEO S2 ReCTA harvested 30 days after its heterotopic implantation. The graft had strong mechanical properties, enabling it to bear compression.
