# Supplementary material for: Pre-epithelialized cryopreserved tracheal allograft for neo-trachea flap engineering
Source: Front Bioeng Biotechnol. 2023 May 5;11:1196521. doi: 10.3389/fbioe.2023.1196521 (PMC10198577; doi:10.3389/fbioe.2023.1196521)
Supplement: Supplementary file 1 [file DataSheet1.PDF]

Pre-epithelialized cryopreserved tracheal allograft for neo-trachea flap engineering

Ning Zeng[1,2,#], Youbai Chen[1,3,#], Yewen Wu[1], Mengqing Zang[1,4], Rene D. Largo[1], Edward I. Chang[1], Mark V. Schaverien[1], Peirong Yu[1]* and Qixu Zhang[1]*

[1]Department of Plastic Surgery, The University of Texas MD Anderson Cancer Center, Houston, TX 77030, USA

[2]Current address: Plastic Surgery Department, Tongji Hospital, Tongji Medical College, Huazhong University of Science and Technology, Wuhan 430070, China

[3]Current address: Department of Plastic and Reconstructive Surgery, Chinese PLA General Hospital, Beijing 100041, China

[4]Current address: Department of Plastic Surgery, Plastic Surgery Hospital, Chinese Academy of Medical Sciences and Peking Union Medical College, Beijing 100041, China

[#]Equal contributions and share first authorship.

***Co-corresponding authors**

Peirong Yu, MD, FACS, Department of Plastic Surgery, Unit 1488, The University of Texas MD Anderson Cancer Center, 1515 Holcombe Blvd., Houston, TX 77030, USA. Tel: +1 713-794-1247; Fax: +1 713-563-8041. E-mail: peirongyu@mdanderson.org

Qixu Zhang, MD, PhD, Department of Plastic Surgery, Unit 602, The University of Texas MD Anderson Cancer Center, 1515 Holcombe Blvd., Houston, TX 77030, USA. Tel: +1 713-563-7565; Fax: +1 713-563-0321. E-mail: qzhang5@mdanderson.org

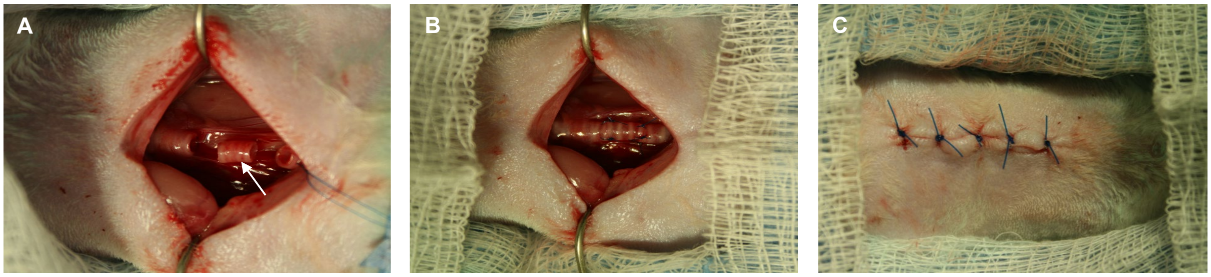

Supplementary Figure 1. Orthotopic implantation of a tracheal graft. (A) A 4-ring tracheal defect was made in the neck of the recipient rat. The white arrow indicates the 4-ring tracheal graft. (B) The tracheal graft was implanted and anastomosed with recipient trachea. (C) The incision was primarily closed

		score	definition
epithelium		0	intact, no loss
		1	less than 30% loss
		2	34-67% loss
		3	68-100% loss
luminal obliteration		0	no obstrution
		1	less than 30% obstruction
		2	34-67% obstrution
		3	68-100% obstruction
cartilage		0	no necrosis
		1	1-33% necorsis
		2	34-67% necrosis
		3	68-100% necrosis

Supplementary Table 1. Tracheal graft necrosis score
